# A Picture Is Worth a Thousand Words: Improving Efficiency in Teledermatology Triage of Skin Cancer Referrals

**DOI:** 10.7759/cureus.105524

**Published:** 2026-03-19

**Authors:** Niall O'Hara, Sufyan Ajmal

**Affiliations:** 1 Plastic and Reconstructive Surgery, Royal Stoke University Hospital, Stoke-on-Trent, GBR

**Keywords:** dermatology, plastic surgery, service improvement project, skin cancer, telemedicine

## Abstract

Rising two-week-wait skin cancer referrals have increased pressure on hospital services. We audited our teledermatology service and evaluated the impact of introducing a standardised referral proforma and clinical photography checklist. This intervention successfully reduced patients’ time to discharge by 35%, decreased face-to-face consultations from 54% to 13% and improved direct-to-surgery referrals all while maintaining clinical safety and improving patient satisfaction. Our findings demonstrate that regular audit and structured interventions in referral pathways can enhance efficiency, reduce unnecessary hospital attendances and support the delivery of a sustainable, modern skin cancer service.

## Introduction

Skin cancer referrals via the two-week-wait pathway continue to increase across the UK, placing significant strain on dermatology and plastic surgery services [1]. Non-melanoma skin cancer represents the most common type of cancer in the UK, with around 250,000 new cases annually, while melanoma accounts for around 17,000 cases each year [2]. Delays in diagnosis and treatment of skin cancer are associated with increased tumour size and stage, more complex reconstructive requirements, reduced patient satisfaction and poorer patient outcomes [3].

Teledermatology has emerged as a key strategy to improve access, streamline triage and reduce unnecessary hospital attendances, particularly for lesions that can be either safely discharged or booked directly for biopsy/excision [4]. The National Health Service (NHS) 2022/23 Priorities and Operational Planning Guidance supports the use of teledermatology to facilitate sufficient diagnostic and treatment capacity and optimise resource allocation [5]. Evidence suggests that teledermatology can provide accurate triage comparable to in-person assessment, while significantly reducing patient waiting times [6].

The effectiveness of teledermatology, however, is critically dependent on the quality of clinical information and imaging provided at referral. Suboptimal referrals may include incomplete histories, poorly described lesions or inadequate information on patients’ comorbidities and mobility, while clinical photographs can also be of varying quality with camera flash artefact, poor lighting or unclear demonstration of the suspect lesion when multiple lesions are present nearby. This can limit clinician confidence in remote decision-making and lead to unnecessary face-to-face appointments, inefficient use of specialist resources or even a risk of wrong-site surgery. The British Association of Dermatologists recommends an annual audit of teledermatology services in order to identify these issues and improve local practice [7].

## Materials and methods

This study evaluated the teledermatology triage service for suspected skin cancer referrals in our busy regional plastic surgery and dermatology department. We assessed the impact of introducing a standardised referral proforma alongside a clinical photography checklist.

Data were collected retrospectively for all skin cancer two-week-wait referrals made between January and June 2024 at the Royal Stoke University Hospital (University Hospitals of North Midlands NHS Trust) in Stoke-on-Trent, UK. The project was registered with and approved by the Trust Audit Department. Our intervention was introduced in March 2024. Two groups of 200 referrals were randomly selected from the pre- and post-intervention periods using a random number generator. The minimum required sample size was calculated using a priori power analysis based on a paired t-test for continuous outcomes with α = 0.05 and power = 80%. All referrals were considered for inclusion. The only exclusion criteria were incomplete records or patients who self-discharged from the service before diagnosis and management.

Our standardised referral proforma included mandatory documentation of the anatomical location of the lesion, symptoms, duration, any past personal or family history of skin cancer, patient risk factors and past medical history relevant to surgery in a minor operating theatre, such as allergies, use of anticoagulation or presence of a pacemaker.

**Figure 1:**
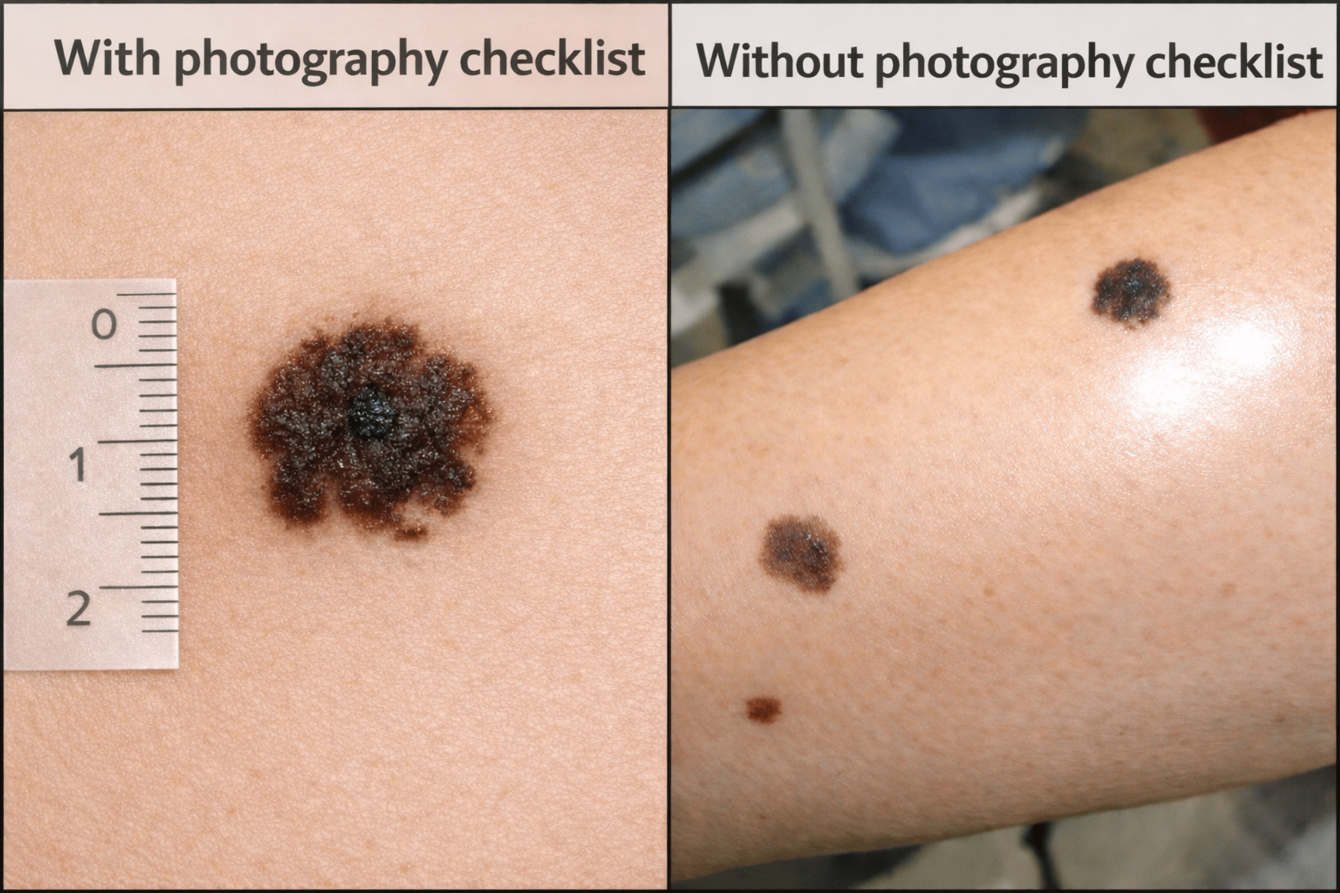
Clinical photography examples

Our clinical photography checklist included taking at least one in-focus photograph in the regional, macroscopic and dermoscopic views, having clear but not harsh lighting, avoiding flash artefact, a plain coloured, ideally white background, use of a ruler to demonstrate size, and clearly labelling the lesion where there are other distracting skin changes nearby. We also mandated having photographs taken by a suitably qualified individual with a postgraduate certificate in clinical photography or equivalent. Figure 1 demonstrates an example of photography taken with and without the checklist.

Outcomes, including time from referral to discharge (either by having surgical biopsy/excision performed, referral on to another low-risk pathway or discharge back to community with advice) and the proportion of patients requiring subsequent face-to-face review, were evaluated, and statistical analysis was performed to assess changes in service efficiency. IBM SPSS Statistics version 29.0 software package (IBM Corp., Armonk, NY) was used for all statistical analyses.

## Results

A total of 4,519 referrals were made over the six-month study period. The most common diagnoses were seborrhoeic keratosis (27%), actinic keratosis (17%) and basal cell carcinoma (15%). The datasets pre- and post-intervention were found to be similar, with a similar proportion of lesions found on review to be benign or malignant.

Compliance with the standardised referral proforma was found to be excellent, and the quality of clinical photographs was reported by the clinical body to have substantially improved. Consultants reported improved clarity in referrals, increased confidence in remote decision-making and fewer cases requiring additional information or default in-person review.

Before the introduction of our proforma, 54% of referrals went on to be seen in a face-to-face clinic to clarify clinical history/examination further in order to aid diagnosis and decision-making. After our intervention, this reduced to just 13%. Similarly, prior to our intervention, only 15% of referrals were able to be confidently returned to community hubs/general practitioners with advice and guidance, compared to 48% after the intervention. Of the referrals, 31% met a sufficient threshold to reach a diagnosis with enough clinical information about the patient’s general health to be able to be referred directly for biopsy or excision in a minor procedure room on a direct-to-surgery pathway without needing a prior appointment, compared to 39% after the intervention. We did note inter-clinician variability in discharge rates (between 16% and 54%), which, on further analysis, was found to be due to individual clinician preferences rather than concern over the quality of the referral.

**Figure 2:**
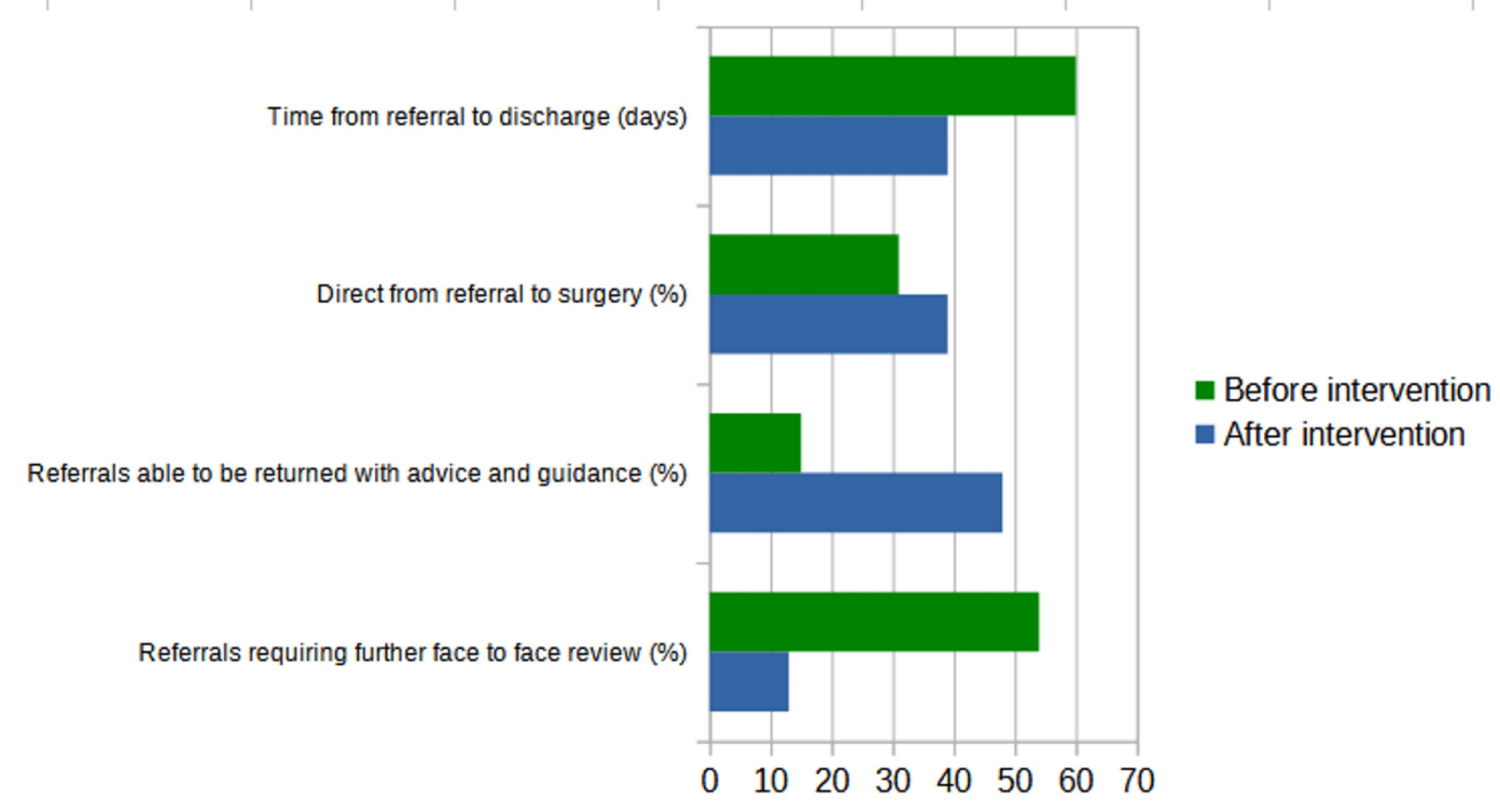
Results

Following the introduction of the standardised referral proforma, time from referral to discharge decreased by 35% from an average of 60 days to 39 days. We also noted a 15% reduction in the number of biopsies being required, indicating greater confidence in clinically diagnosing lesions as benign. Figure 2 demonstrates these results before and after the introduction of our proforma.

On a patient satisfaction survey of the new service, 91% of patients reported being satisfied with the use of teledermatology to triage their referral. Comments ranged from feeling “reassured,” appreciating the “rapid access to a specialist” and being “impressed with the use of modern technology.” Consultants estimated that analysis of each teledermatology referral took on average 90 seconds compared to 10-minute face-to-face clinic appointments. If scaled for the 4,519 referrals in our six-month period, this would result in a saving of 1,280 hours or 320 four-hour consultant programmed activities per year.

## Discussion

This study demonstrates that teledermatology can effectively reduce waiting list times while safely managing a substantial proportion of two-week-wait skin cancer referrals. The introduction of our structured referral proforma and clinical photography checklist significantly improved service efficiency, reducing both time to discharge and unnecessary face-to-face attendances and facilitating direct-to-surgery pathways. The reduction in face-to-face appointments has implications for patient convenience, clinic capacity, environmental impact and resource utilisation [8]. Faster discharge decisions also support pathway targets and reduce patient anxiety [9].

The high proportion of benign lesions underscores the need for continued primary care education in skin lesion assessment and risk stratification [10]. Improved referral quality also enhances clinician confidence and may reduce over-treatment and unnecessary procedures [11].

Several limitations must be acknowledged. First, this was a single-centre study, which may limit generalisability to other healthcare settings with different referral pathways, patient demographics or resource availability. Second, the study relied on service-level outcomes such as time to discharge and face-to-face consultations rather than long-term patient-centred outcomes, including recurrence rates, survival or quality of life measures. Third, the retrospective design may introduce selection bias, despite random sampling of referrals. Fourth, inter-clinician variability in discharge decisions highlights that subjective clinical judgement still influences outcomes, even with standardised tools.

Our intervention requires trained clinical photographers and engagement from referring clinicians. Therefore, scaling this intervention to other centres may be limited by the availability of personnel, training resources or funding. Other potential barriers to rolling out similar services in other units include resistance to the introduction of any new system, ensuring ongoing community team engagement and adequate job planning to allow time for consultants to triage teledermatology referrals. Some patients also wanted to retain the option of seeing a specialist in person. However, overall, in our cohort, we encountered very few barriers. Our changes were well received by patients and well supported and funded by the local clinical commissioning group.

## Conclusions

Standardised teledermatology referrals incorporating structured referral proformas and clinical photography significantly improve efficiency, reduce unnecessary face-to-face consultations and maintain clinical safety within two-week-wait skin cancer pathways. This low-cost intervention provides a simple, reproducible and scalable model for modern skin cancer service delivery and supports broader adoption of teledermatology to enhance patient care and resource utilisation.
